# Kinetics of functionalised carbon nanotube distribution in mouse brain after systemic injection: Spatial to ultra-structural analyses

**DOI:** 10.1016/j.jconrel.2015.12.039

**Published:** 2016-02-28

**Authors:** Julie T.-W. Wang, Noelia Rubio, Houmam Kafa, Enrica Venturelli, Chiara Fabbro, Cécilia Ménard-Moyon, Tatiana Da Ros, Jane K. Sosabowski, Alastair D. Lawson, Martyn K. Robinson, Maurizio Prato, Alberto Bianco, Frederic Festy, Jane E. Preston, Kostas Kostarelos, Khuloud T. Al-Jamal

**Affiliations:** a Institute of Pharmaceutical Science, Faculty of Life Sciences & Medicine, King's College London, London SE1 9NH, UK; b CNRS, Institut de Biologie Moléculaire et Cellulaire, Laboratoire d'Immunopathologie et Chimie Thérapeutique, Strasbourg F-67000, France; c Dipartimento di Scienze Chimiche e Farmaceutiche, Università di Trieste, Trieste 34127, Italy; d Centre for Molecular Oncology, Barts Cancer Institute, Queen Mary University of London, London EC1M 6BQ, UK; e UCB Celltech, Slough, Berkshire SL1 3WE, UK; f Tissue Engineering and Biophotonics, Dental Institute, King's College London, London SE1 9RT, UK; g Nanomedicine Laboratory, UCL School of Pharmacy, University College London, Brunswick Square, London, UK

**Keywords:** Carbon nanotubes, Brain drug delivery, Blood–brain barrier, Nanomedicine, SPECT/CT imaging, Multi-photon luminescence microscopy

## Abstract

Earlier studies proved the success of using chemically functionalised multi-walled carbon nanotubes (*f*-MWNTs) as nanocarriers to the brain. Little insight into the kinetics of brain distribution of *f*-MWNTs *in vivo* has been reported. This study employed a wide range of qualitative and quantitative techniques with the aim of shedding the light on *f*-MWNT's brain distribution following intravenous injection. γ-Scintigraphy quantified the uptake of studied radiolabelled *f*-MWNT in the whole brain parenchyma and capillaries while 3D-single photon emission computed tomography/computed tomography imaging and autoradiography illustrated spatial distribution within various brain regions. Raman and multiphoton luminescence together with transmission electron microscopy confirmed the presence of intact *f*-MWNT in mouse brain, in a label-free manner. The results evidenced the presence of *f*-MWNT in mice brain parenchyma, in addition to brain endothelium. Such information on the rate and extent of regional and cellular brain distribution is needed before further implementation into neurological therapeutics can be made.

## Graphical abstract

**Figure:**
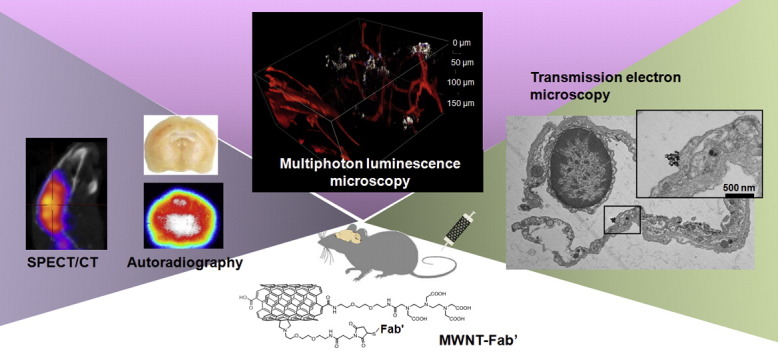

## 1 Introduction

Drug transfer to the brain is greatly limited at the blood–brain barrier (BBB) by highly restrictive tight junctions between capillary endothelial cells [1]. Improvements in drug transport across the BBB have been shown by directly altering the physicochemical properties of the drug, or coupling drugs to a vector including peptides, antibodies or nanoparticles (NPs) [2], [3] which are transported across the BBB by endogenous receptor- or adsorptive-mediated transcytotic pathways.

Delivery systems including liposomes [4], polymeric nanoparticles [5], [6], [7], gold nanoparticles [8], and to a lesser extent, dendrimers [9], [10], have raised tremendous hope for the treatment of several central nervous system (CNS)-related disorders [11], [12]. The maturation of nanotechnology applications in biomedicine means that NPs with high drug payloads can be engineered to have physiochemical properties favourable for brain uptake. Furthermore, they can be surface functionalised with biological ligands to target or cross the BBB.

Carbon nanotubes (CNTs) are a rather new category of nanomaterials and have received great attention in the biomedical field [13], [14]. We have developed biocompatible chemically functionalised CNTs (*f*-CNTs) [15], [16] and investigated their ability as drug carriers, gene carriers or as imaging agents *in vivo* [17], [18], [19]. We demonstrated that *f*-CNTs improve the anti-apoptotic effect of Caspase-3 siRNA after focal stroke in rats when the *f*-CNT:siCas 3 complex is injected cortically [20] and that functionalisation is important for cell uptake [21]. We recently reported the intrinsic ability of *f*-MWNT to trancytose across an *in vitro* BBB co-culture model with preliminary evidence of accessing mice brain following i.v. injection [22]. In another study, we compared the *in vivo* biodistribution profiles of two series of functionalised multi-walled CNTs (*f*-MWNTs) following tail vein injection in mice [23]. The “thin” *f*-MWNT, conjugated to humanised IgG or fragment antigen binding region (Fab′), exhibited higher brain affinity compared to the other *f*-MWNT conjugates in the study. The present study aims to understand the rate and extent of passive *f*-MWNT's access to brain parenchyma, using a panel of quantitative and qualitative techniques, ranging in resolution from “macro” to “nano” scales. The thin *f*-MWNT-Fab′, previously showing highest brain uptake, was used as a prototype. Techniques used include quantitative γ-scintigraphy, 3D-single photon emission computed tomography/computed tomography (SPECT/CT) imaging and autoradiography, which altogether provided quantitative, temporal and spatial information of high sensitivity. Direct imaging of *f*-MWNT-Fab′ in brain tissue was carried out using Raman spectroscopy and multi-photon luminescence microscopy, coupled with lifetime imaging, offering excellent spatial resolution. Transmission electron microscopy (TEM) also revealed the presence of *f*-MWNT-Fab′ in brain parenchyma at an ultra-structural scale.

## 2 Materials and methods

### 2.1 Materials

Reagents for CNT functionalisation and other chemicals were purchased from Sigma-Aldrich or Acros Organics (UK) if not specified below. The MWNTs were provided by Nanocyl (batch# 171119, Belgium). MWNTs were produced by the catalytic carbon vapour deposition process with above 95% purity. Antibody hCTM01 Fab′ against human polymorphic epithelial mucin (MUC1) was obtained from UCB Celltech (UK). The radioactive probe [^111^In]Cl_3_ was purchased from Mallinckrodt Pharmaceuticals (The Netherlands) as an aqueous solution in 0.5 M HCl and used without further purification. Instant thin layer chromatography paper impregnated with silica gel (iTLC-SG) was obtained from Agilent Technologies (UK). Calcium fluoride (CaF_2_) slides (0.5 mm thickness, 20 mm diameter) for Raman spectroscopy were supplied by Crystran Ltd. (UK). Mini protease inhibitor cocktail kits (Roche Diagnostics, USA) was used to prepare brain lysates for BCA protein assay (Pierce BCA kit, Thermal Fisher Scientific, UK), Western blotting and ELISA assay (BD OptEIA™, Mouse TNF ELISA set, BD Biosciences, UK). 1,1′-Dioctadecyl-3,3,3′,3′-tetramethylindocarbocyanine perchlorate (DiI) was purchased from Santa Cruz Biotechnology Inc. (Germany). Minimum Essential Medium (MEM) containing 25 mM HEPES for preparation of isolated brain capillaries was purchased from Life Sciences (UK).

### 2.2 Synthesis of chemically functionalised MWNTs

The synthesis of chemically functionalised MWNTs has been described previously [23]. In brief, carboxylated and shortened MWNTs were prepared by oxidation using strong acids. Following 1,3-dipolar cycloaddition and amidation reactions, MWNTs were functionalised with diethylenetriaminepentaacetic (DTPA) dianhydride chelating molecules for complexation of the nuclear imaging probe ^111^In and further modified with a model humanised MUC1 antibody fragment antigen binding region (Fab′).

### 2.3 Radiolabelling of *f*-MWNTs

The DTPA conjugated *f*-MWNT-Fab′ was radiolabelled with ^111^In as described previously [23]. Briefly, MWNT-Fab′-DTPA (0.5 mg/ml in 5% dextrose) was dispersed by bath sonication and then buffered with an equal volume of 0.2 M ammonium acetate solution (pH 5.5) containing ^111^InCl_3_ (30 MBq or 4 MBq per 100 μg [^111^In]MWNT-Fab′ for SPECT imaging or γ-counting respectively). Solutions were mixed thoroughly by vortexing every 5 min for 30 min at room temperature. The reaction was stopped by the addition of 0.1 M EDTA chelating solution (1/20, v/v). ^111^InCl_3_ alone was also subjected to the same conditions as a control. The labelling efficiency was analysed by TLC. [^111^In]MWNT-Fab′ solutions were spotted onto TLC strips, developed in 0.1 M ammonium acetate buffer containing 50 mM EDTA as a mobile phase and allowed to dry before analysis. The auto-radioactivity of each strip was developed and counted quantitatively using a Cyclone phosphor detector (Packard Biosciences). Excess unreacted ^111^In was removed by centrifugation at 13,000 rpm for 30 min to pellet [^111^In]MWNT-Fab′, with unreacted ^111^In remaining in the supernatant. The pellets were resuspended in 5% dextrose at 0.5 mg/ml for injection. TLC was carried out prior to each injection to ensure the absence of free ^111^In in the solution.

The stability of radiolabelling was examined by incubating dispersion of [^111^In]MWNT-Fab′ with an equal volume of PBS or human serum at 37 °C for 24 h. The results were also analysed by TLC.

### 2.4 Animals

All *in vivo* experiments were conducted under the authority of project and personal licences granted by the UK Home Office and the UKCCCR Guidelines (1998). Female C57/Bl6 mice aged 6–8 weeks were purchased from Harlan (UK) for all the experiments.

### 2.5 Organ biodistribution by γ-scintigraphy

To study the organ uptake including brain, C57/Bl6 mice were intravenously (i.v.) injected with 50 μg of [^111^In]MWNT-Fab′ (0.5 mg/ml in PBS) *via* a tail vein. At 5 min, 30 min, 1 h, 4 h or 24 h after injection, blood samples were collected from a tail vein, the body was perfused with 40 ml heparinised saline (50 U/ml) through the left ventricle of the heart to wash out MWNTs remaining in blood or loosely bound to tissues and the brain and other major organs were rapidly excised. Excised brain and other tissues were weighed and the radioactivity was measured by γ-scintigraphy (LKB Wallac 1282 Compugamma, PerkinElmer).

### 2.6 Analysis of the uptake in brain parenchyma and capillaries by capillary depletion

To separate the brain capillaries from the brain parenchyma, the capillary depletion method modified for mice [24], [25] was performed. After γ-counting, the whole brain was homogenised in ice-cold physiological buffer (10 mM HEPES in HBSS, pH 7.4) using a glass homogeniser on ice. When brain tissues were homogenised with 10 strokes of the pestle in the homogeniser, a dextran solution (M.W. 64–76 kDa, 26%) was added for a further brief homogenisation step (3 strokes of the pestle). The homogenates were centrifuged at 5400 ×* g* for 15 min at 4 °C. The supernatants containing the brain parenchyma and the pellets containing the brain capillaries were separately collected, followed by γ-counting of the radioactivity of each fraction. The weight of the brain parenchyma was estimated as the weight of the whole brain, as the weight of capillaries is minimum compared to parenchyma.

### 2.7 TNF-α assay by ELISA

Mice were i.v. injected with 50 μg of MWNT-Fab′-DTPA (0.5 mg/ml in PBS) and the brains sampled at 24 h and 72 h after injection. Brain tissues were kept frozen at − 80 °C until further analyses. Brain tissues were homogenised in ice-cold RIPA lysis buffer (150 mM NaCl, 0.1% Triton X-100, 0.5% sodium deoxycholate, 0.1% SDS, 50 mM Tris–HCl, pH 8) containing protease inhibitor. Homogenates were centrifuged at 17,949 ×* g* for 30 min and the supernatants were collected to measure the protein concentration and TNF-α levels using for BCA and ELISA assays respectively. Both assays were carried out according to their manufacturers' instruction.

### 2.8 The effect of dexamethasone pre-treatment on brain uptake

To investigate if there was MWNT-induced inflammation that may have affected its brain uptake, mice received a pre-treatment of dexamethasone (10 mg/kg, i.p.) and were then given an injection of [^111^In]MWNT-Fab′ (50 μg, 0.5 mg/ml in PBS, i.v.), an hour later. Dexamethasone was prepared in dimethyl sulfoxide at 50 mg/ml and then diluted in saline for injection. At 1 h post [^111^In]MWNT-Fab’ injection, mice were perfused with 4% paraformaldehyde (PFA) and brains were isolated for gamma counting.

### 2.9 Brain 3D SPECT/CT imaging

*In vivo* brain uptake of [^111^In]MWNT-Fab′ was also examined by 3D whole body SPECT/CT imaging. Mice were anaesthetised by isoflurane inhalation and i.v. injected with 50 μg of [^111^In]MWNT-Fab′ (0.5 mg/ml in PBS). SPECT/CT scans were carried out at multiple time points on the same animal, immediately post injection, at 4 h and 24 h in the prone position using the Nano-SPECT/CT scanner (Bioscan, USA). SPECT scanning was acquired over 24 projections (60 s per projection), using a 4-head scanner with 1.4 mm pinhole collimators, for a total acquisition time of 30–40 min. CT scans were performed using a 45 kVP X-ray source after each SPECT. SPECT images were reconstructed using HiSPECT (Scivis GmbH, Germany) and CT images were reconstructed using InVivoScope™, proprietary Bioscan software. Both images were fused and analysed by InVivoScope™.

### 2.10 Autoradiography

To investigate the regional distribution of *f*-MWNTs in the brain, [^111^In]MWNT-Fab′ (5–7 MBq, 50 μg, 0.5 mg/ml in PBS) was i.v. injected in mice and the brains were isolated at 5 min, 4 h or 24 h after injection for autoradiography. Studies using mice injected with [^111^In]EDTA containing similar radioactivity (5–7 MBq) were also performed as a control for comparison. Each brain was cut into 2 mm thick coronal sections using a mouse brain matrix (Zivic-Miller, USA). Sections were placed between two glass microscope slides and these sandwich units were in contact with super sensitive plates (Cyclone® storage phosphor screen, Packard) for 18–20 h in autoradiography cassettes (Kodak Biomax Cassette®) before scanning using cyclone phosphor detector (Packard Biosciences).

### 2.11 Administration of *f*-MWNTs using *in situ* brain perfusion

[^111^In]MWNT-Fab′ was administered to mouse brains using the *in situ* brain perfusion technique *via* cardiac perfusion [26]. This protocol was carried out to maintain a steady blood concentration of [^111^In]MWNT-Fab′ at all times and administer [^111^In]MWNT-Fab′ to brain directly *via* carotid and vertebral arteries rather than *via* intravenous injection, so avoiding first pass effect of peripheral organs. Mice were weighed and anaesthetised with i.p. injection of a cocktail solution of Domitor® (Orion Pharma, Finland) and Vetalar™ V (Pfizer, UK), freshly mixed before injection. [^111^In]MWNT-Fab′ in PBS (0.5 mg/ml) was added to the perfusion solution, a protein containing Ringer's buffer (117 mM NaCl, 4.7 mM KCl, 2.5 mM CaCl_2_, 1.2 mM MgSO_4_, 24.8 mM NaHCO_3_, 1.2 mM KH_2_PO_4_, 10 mM glucose, and 30 g/l bovine serum albumin). During perfusion, pre-gassed (5% CO_2_ in O_2_) Ringer's buffer containing [^111^In]MWNT-Fab′ (0.4 μg/ml) was maintained at 37 °C using a water heater and passed through a filter and bubble trap before reaching the left ventricle of the heart. Perfusion rate was kept constant at 5 ml/min using a peristaltic pump. Mice were perfused for 5, 10 or 30 min followed by one minute perfusion without [^111^In]MWNT-Fab′ to wash out residual construct from the vasculature. At the end of perfusion, different brain compartments (*i.e.* choroid plexus, brain cortex, olfactory bulbs, cerebellum, thalamic region and brainstem) were dissected and weighed, and the radioactivity from each sample was measured by γ-scintigraphy (LKB Wallac 1282 Compugamma, PerkinElmer, UK).

### 2.12 Histology studies

For histological examination, mice were i.v. injected with a dose of MWNT-Fab′-DTPA at 200 μg/mouse (1 mg/ml in PBS). Considering the obtained % ID in brain, a higher injection dose was given here, as well as in the following Raman, multi-photon and TEM studies, to facilitate the detection of MWNT-Fab′-DTPA microscopically. Brains were harvested at 5 min after cardiac perfusion with 4% PFA for general histological examination. Samples were then wax-embedded and sectioned for Nissl and Luxol Fast Blue or Neutral Red stains according to standard histological protocols at the Royal Veterinary College, UK. Stained sections were analysed using a Leica DM 1000 LED Microscope (Leica Microsystems, UK) coupled with CCD digital camera (Qimaging, UK).

### 2.13 *Ex vivo* Raman spectroscopic analysis

Raman spectroscopy studies of MWNT-Fab′-DTPA in the mouse brain were performed using an inVia™ Raman microscope (Renishaw, UK). Spectra were acquired in the StreamLine™ scanning mode. Mice were either i.v. injected with MWNT-Fab′-DTPA (200 μg, 1 mg/ml in PBS) or saline (PBS) and the brains were isolated at 5 min after injection. Both frozen brain sections and isolated brain capillaries were prepared for Raman spectroscopy. For frozen sections, the entire brain was snap-frozen and sectioned into 10 μm thick sections. For the isolated brain capillaries, freshly isolated brains were homogenised in MEM containing 25 mM HEPES. The homogenates were then filtered using a 60 μm nylon mesh to separate the capillaries from the homogenates. The capillaries were suspended in MEM and further concentrated by centrifugation at 3220 *g* for 15 min. Capillaries or brain sections were deposited on a calcium fluoride slide and allowed to dry. MWNT-Fab′-DTPA in DMF dispersion (50 μl, 20 μg/ml in DMF), deposited on a calcium fluoride (CaF_2_) slide, was used as a reference. Samples were scanned using a 785 nm HPNIR785 diode laser (15 mW, 10% laser power and 10 s exposure) through a 60 ×/1.20 NA water objective. Raman signals were acquired using a 300 lines/mm grating centred between 917.67 cm^− 1^ and 2326.15 cm^− 1^ and 10 s CCD exposure time. The spectral resolution was 8.55 cm^− 1^. For each sample, 3 regions (119 μm × 119 μm each) were imaged and around 40,000 spectra per region were recorded. Pearson-based cluster analysis of the data set was performed using an in-house programme [27] designed to identify independent cluster components in the sample.

### 2.14 Multi-photon luminescence and lifetime microscopy

Mice were i.v. injected with MWNT-Fab′-DTPA (200 μg, 1 mg/ml in PBS). At 1 h after injection, mice were transcardially perfused consecutively with PBS, DiI solution (0.12 mg/ml in PBS–5% glucose mixture) and 4% PFA [28]. Brains were sectioned into 1 mm thick slices and kept in 4% PFA in the dark at 4 °C until use. Brain slices were placed onto microscope slides and covered with coverslips for microscopic imaging. Multi-photon luminescence images of MWNT-Fab′-DTPA and DiI in brain were acquired with excitation at 950 nm (~ 140 fs pulse width, 80 MHz) using a tunable Coherent Chameleon II Ti:Sapphire IR laser (Coherent, USA). Imaging was carried out using a confocal microscope (A1R Si MP Confocal, Nikon, Japan) with a 40 × water immersion objective (Nikon, Japan) and fluorescence signals were captured *via* three bandpass filters (492 nm SP, 525/50 nm, and 575/25 nm appearing in blue, green and red respectively). Fluorescence from DiI and MWNT-Fab′-DTPA could be differentiated since DiI fluorescence was mainly detected in the red channel while MWNT-Fab′-DTPA signals were measured in all channels.

Lifetime microscopy was further carried out on the physically-mixed dispersion of DiI and MWNT-Fab′-DTPA, as well as the brain slices from the above two-photon microscopy studies. This imaging technique can distinguish signals from DiI and MWNT-Fab′-DTPA with good resolution due to the distinct differences in their lifetimes. The DiI & MWNT-Fab′-DTPA mixture was prepared by mixing equal volumes of DiI (1 μg/ml in ethanol) and MWNT-Fab′-DTPA (1 mg/ml in H_2_O). A drop of the mixture (20 μl) was deposited onto a microscope slide and allowed to dry for imaging. An in-house multi-photon imaging system built onto a Nikon Eclipse Ti inverted microscope (Nikon, Tokyo, Japan) was employed. Samples were excited using a tunable Ti:sapphire laser (~ 140 fs, 80 MHz, Coherent, USA) at 950 nm and no confocal pinhole was used. Lifetime imaging and intensity data were acquired using a Becker & Hickl HPM-100-40 high speed hybrid detector and a SPC-150 TCSPC card (Becker & Hickl, Germany) *via* a 20 ×/0.75NA Nikon objective lens. Lifetime data was analysed using an in-house software.

### 2.15 Transmission electron microscopy studies

Mice were i.v. injected with MWNT-Fab′-DTPA (200 μg, 1 mg/ml in PBS) and the brains were isolated at 5 min after whole body cardiac perfusion with freshly-prepared 50 ml of 2.5% glutaraldehyde solution in 0.1 M cacodylate buffer. The brains were immersed in the perfusate for 24 h at 4 °C before taking 1 mm thick sections using a mouse brain matrix (Zivic-Miller, USA). Sections were rinsed with 0.1 M cacodylate buffer to remove excess glutaraldehyde and kept in 0.1 M cacodylate buffer at 4 °C until use. The brain sections were stained with 1% osmium tetroxide for 3 h, dehydrated with increasing concentrations of ethanol from 70% to 100%, and then soaked with propylene oxide for 1 h to facilitate the brain infiltration of embedding resin. Brain sections were incubated with a solution of 1:1 propylene oxide: epoxy resin (43.7% Araldite CY212, 54.5% DDSA and 1.7% DMP30) for 24 h at room temperature with shaking. Finally, brain sections were embedded into epoxy resin for 3 days at 60 °C, allowing the polymerisation process to occur. Ultrathin sections (65 nm) were cut on a 45° diamond knife (DiATOME, USA) from the blocks using a microtome (Leica Richert Ultracut) and collected on copper 200-mesh grids. Some brain sections were further stained with uranyl acetate (25% in methanol) for 3 min and 3% lead citrate solution for 8 min to visualise cells with better image contrast (Fig. S5).

TEM analysis was also carried out on brain capillaries extracts. The brain capillaries from untreated mice and mice injected with MWNT-Fab′ were isolated following the same procedures as described above. The collected pellets containing brain capillaries were re-suspended in 2.5% glutaraldehyde solution in 0.1 M cacodylate buffer for fixation for 24 h at 4 °C. Samples were washed with 0.1 M cacodylate buffer by centrifugation at 3220 *g* for 15 min and the pellets were re-suspended in 50 μl of 1% molten agarose and allowed to solidify at room temperature. The capillaries in agarose gel were stained with 1% osmium tetroxide for 3 h, processed as described above and then imaged by TEM.

Bright field TEM images of brain sections and isolated capillaries were acquired using a Philips CM12 transmission electron microscope (Phillips, The Netherlands) at 80 kV acceleration voltage, with 150 μm objective aperture.

### 2.16 Statistical analysis

Quantitative results are expressed as mean ± S.D. (n = 3–4). Statistical significance was calculated with one-way ANOVA. Significant difference was established at *p* < 0.05.

## 3 Results and discussion

### 3.1 Synthesis, radiolabelling and stability studies

**Fig. 1:**
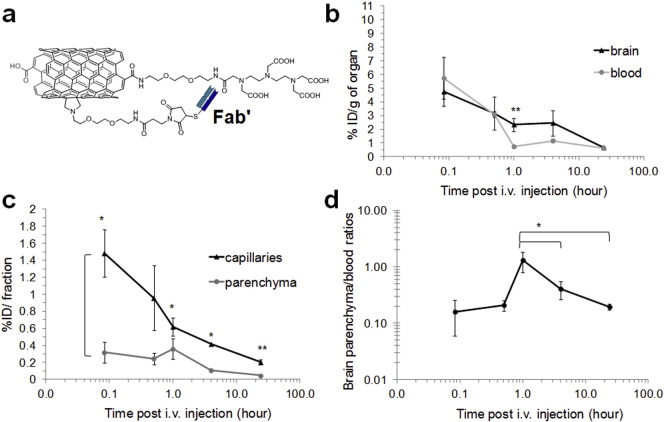
Blood circulation and brain uptake of [^111^In]MWNT-Fab′ after i.v. injection at different time points by γ-scintigraphy. (a) The structure of chemically functionalised MWNT-Fab′-DTPA; (b) % ID/g of whole brain and % ID/g of blood; (c) disposition of [^111^In]MWNT-Fab′ (% ID) in brain capillaries and parenchyma after capillary depletion; (d) brain parenchyma to blood ratio calculated from the concentration of [^111^In]MWNT-Fab′ in brain parenchyma and blood (% ID/g). Mice were i.v. injected with 50 μg of [^111^In]MWNT-Fab′. Results are expressed as mean ± S.D. (n = 4). Statistical analysis was performed using One-Way ANOVA (*p *<* 0.05; **p ≤ 0.01).

Chemical synthesis and detailed physiochemical characterisation of the MWNT-Fab′-DTPA conjugate have been assessed previously (Fig. S1) [23]. The results from several analyses such as Kaiser test, thermogravimetric analysis, gel electrophoresis and surface plasma resonance determined the antibody loading, assured the antibody activity after conjugation and all confirmed the success of functionalisation. No structure changes before and after antibody conjugation were observed using TEM [23]. The diameter and length of MWNT-Fab′ DTPA were measured as 9.2 ± 2.7 nm and 396 ± 290, respectively, with the Fab′ loading as 3 μmole of Fab′/g of CNT. The chemical structure of the studied MWNT-Fab′-DTPA is shown in Fig. 1a and Fig. S1. MWNT-Fab′ functionalised with DTPA was radiolabelled with ^111^In to yield [^111^In]MWNT-Fab′ and 49.4 ± 5.4% labelling efficiency was achieved as assessed by TLC examination (Fig. S2). Non-chelated ^111^In was removed by centrifugation. [^111^In]MWNT-Fab′ remained stable in 50% serum and PBS at 37 °C for 24 h. Negligible free ^111^In was detected as shown by TLC (Fig. S2).

### 3.2 Quantitative brain uptake analyses by γ-scintigraphy

Mice were i.v. injected with [^111^In]MWNT-Fab′ to assess the brain uptake profiles. The brain and other major organs were excised at multiple time points at 5 min, 30 min, 1 h, 4 h and 24 h for γ-scintigraphy. Transcardial perfusion was performed before harvesting organs in order to wash out *f*-MWNTs still present in the vascular compartment at the time of sacrifice. Blood circulation profiles were also examined by taking blood samples immediately before perfusion.

Fig. 1b illustrates the amount of [^111^In]MWNT-Fab′ in the brain and blood over time, expressed as percentage of injected dose per gram of brain or blood (% ID/g of organ). The brain accumulation was rapid, and could be detected at the earliest time point assessed in this study of 5 min post injection. Brain levels then decreased steadily up to 24 h, in contrast to the relatively rapid drop in blood concentration observed within the first hour after injection. [^111^In]MWNT-Fab′ displayed ~ 5% and 3% ID/g of brain at 5 min and 30 min post-injection (p.i.). At 1 h, a significantly higher brain uptake (2.2 ± 0.6%) compared to blood (0.7 ± 0.1%) was detected (*p* ≤ 0.01). Capillary depletion [24], [25] employed to separate brain parenchyma from the capillaries (Fig. 1c), revealed higher % ID detected in the capillary fraction compared to parenchyma over the studied 24 h, suggesting the high affinity of [^111^In]MWNT-Fab′ to brain endothelium. Interestingly, [^111^In]MWNT-Fab′ levels declined gradually in the capillary fractions, over the first hour, compared to the more sustained profile observed in the brain parenchyma. Fig. 1d compares the concentration in the blood versus brain parenchyma over time. The brain parenchyma to blood ratio represents the extent of brain penetration of [^111^In]MWNT-Fab′, *i.e.* the translocation from blood circulation, crossing brain capillaries, and reaching parenchyma [29]. The highest brain parenchyma (% ID/g) to blood (% ID/g) ratio obtained was 1.31 ± 0.52 at 1 h, which was significantly higher than the values measured at 4 h and 24 h.

The blood circulation and brain uptake of [^111^In]MWNT without Fab′ conjugation were also studied. The results in comparison with [^111^In]MWNT-Fab′ are shown in Fig. S3. Similar blood profiles with short blood circulation time were obtained for [^111^In]MWNT and [^111^In]MWNT-Fab′ (Fig. S3a). However, as shown in Fig S3b, significantly lower brain accumulation was measured for [^111^In]MWNT at earlier time point (*i.e.* 5 min and 30 min post injection) compared to [^111^In]MWNT-Fab′ (*p* < 0.05). After capillary depletion, [^111^In]MWNT also exhibited lower uptake in both brain parenchyma and capillaries (Fig. S3c–d). As a result, lower brain parenchyma to blood ratios were obtained for [^111^In]MWNT compared to [^111^In]MWNT-Fab′ over the studied time course in which the highest ratio was measured as 0.14 ± 0.02 at 4 h post injection.

The introduction of any synthetic particle in the circulation raises concerns about possible inflammation particularly since inflammation can cause increased BBB permeability, thus resulting in high brain uptake [30], [31]. We have reported previously that the *f*-CNTs undergone similar functionalisation induced low levels of pro-inflammatory cytokines with the observation of degradation at 48 h after stereotactic injection [21], [32]. In our recent work, no significant toxicity was measured in primary neurons isolated from different brain regions of foetal rats after incubation with *f*-CNTs for 24 h [33]. Here we examined brain levels of the pro-inflammatory cytokine TNF-α after MWNT-Fab′-DTPA administration by enzyme-linked immunosorbent assay (ELISA). The cytokine levels in brain increased at 24 h post-injection but decreased at 72 h to the levels similar to control brains (Fig. S4a). Although TNF-α increased in brain with *f*-MWNT administration, it was a modest increase and considerably lower than levels in an inflammation model using LPS for example [34]. To further eliminate brain inflammation as a causative factor for brain uptake of *f*-MWNT, mice were pre-treated with dexamethasone (10 mg/kg, i.p.), an anti-inflammatory drug, one hour prior to [^111^In]MWNT-Fab′ injection. [^111^In]MWNT-Fab′ was then injected and the brain uptake was assessed at 1 h post-injection. This is the time point where the highest brain parenchyma/blood ratio was achieved for [^111^In]MWNT-Fab′ (Fig. 1d). No significant difference in brain uptake was detected with or without the pre-treatment of dexamethasone (*p* = 0.4, Fig. S4b). TEM images of brain also showed the maintenance of brain capillaries after i.v. injection of MWNT-Fab′-DTPA. As shown in Fig. S5, capillaries remained intact and were seen in a smooth circular shape, not extruded, with tight junction clearly observed (pointed by the black arrow, Fig. S5a). Taking together, these results indicate neither inflammation nor the damage in BBB (BBB leakage) was induced by the introduction of MWNT-Fab′-DTPA and they were not the reasons for the high brain uptake of MWNT-Fab′-DTPA.

A targeting *f*-CNTs system was developed by Ren et al. for brain glioma therapy [35]. MWNTs were incorporated with an anticancer drug doxorubicin and polyethylene glycol, in which the polymer was conjugated with a brain targeting ligand Angiopep-2. This targeting system demonstrated enhanced brain uptake of doxorubicin. This was confirmed by an indirect detection of doxorubicin fluorescence signals from the construct. In this regard, the present work performed a quantitative assessment of the distribution kinetics of the carrier in the brain. The brain uptake values of ~ 2–5% ID/g reported in this study are considerably higher compared to other types of targeted nanoparticles, which range from 0.3% to 0.5% ID/g of brain [25], [36], [37], [38]. More importantly, the brain parenchyma to blood ratios of [^111^In]MWNT-Fab′ were in excess of 0.2 between 30 min to 24 h post injection and peaked at 1.31 at 1 h. It is noteworthy that many CNS drugs including morphine show good CNS distribution with their brain to blood ratios > 0.5 [39], [40] and most CNS drugs in the market can achieve only 0.2% of their peripheral doses [30]. The studied MWNT-Fab′ clearly demonstrated superior brain uptake and holds a great potential as a delivery vehicle for CNS diseases. Taking into account the studied *f*-MWNT achieved 15–20% antibody/Fab′ loading (μg of antibody/μg of CNT) [23], one can simply assume that this payload can be replaced with the therapeutic antibodies of choice.

The Fab′ used here is against human polymorphic epithelial mucin (MUC1) which is not expressed in mouse brain (Fig. S6). Therefore, the significant brain uptake of [^111^In]MWNT-Fab′ is unlikely to be due to the antibody targeting effect but can be attributed to the nanotube's ability to penetrate endothelial/brain cells.

The biodistribution profiles of [^111^In]MWNT and [^111^In]MWNT-Fab′ in organs other than brain and blood are shown in Fig. S7. Both [^111^In]MWNT and [^111^In]MWNT-Fab′ exhibited prominent uptake in liver, lung and spleen, consistent with the results obtained in our previous studies, conducted without transcardial perfusion [23].

### 3.3 3D SPECT/CT imaging and autoradiography

**Fig 2:**
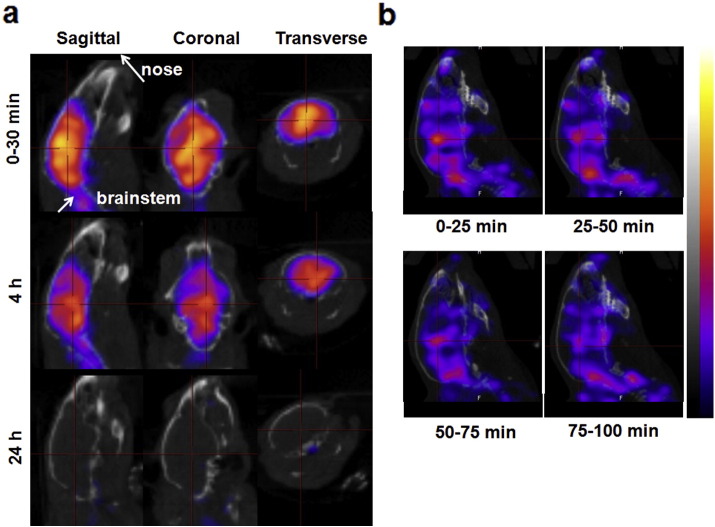
*In vivo* SPECT/CT imaging of mouse brains. (a) Sagittal, coronal and transverse views from 3D reconstructed SPECT/CT images of mouse brains after i.v. injection of [^111^In]MWNT-Fab′ (50 μg, 5–7 MBq) at 0–30 min, 4 h and 24 h post injection; (b) dynamic SPECT/CT imaging of mouse brain (sagittal views) within 100 min after injection of [^111^In]MWNT-Fab′.

The brain uptake of radiolabelled [^111^In]MWNT-Fab′ after i.v. injection was also confirmed by SPECT/CT imaging. In line with the results obtained from γ-scintigraphy, higher signals were detected at early time points (0–4 h) while the radioactivity decreased at 24 h (Fig. 2a). [^111^In]MWNT-Fab′ seemed to distribute over the entire brain but appeared to be more concentrated in the midbrain region, seen most clearly in the 4 h images (indicated by the cross hairs).

Dynamic SPECT/CT imaging of [^111^In]MWNT-Fab′ in the mouse brain was further performed to monitor the distribution changes, particularly at early time points after injection. Mice underwent 4 successive SPECT scanning with images acquired every 25 min for a total of 100 min. Physiological shifting of the radioactivity in the brain could be visualised over time shown in the sagittal views in Fig. 2b. Heterogeneous distribution could be clearly observed using this imaging method. Mice were also injected with the [^111^In]EDTA radiolabel alone as controls and subjected to both γ-scintigraphy and SPECT/CT imaging studies (Fig. S8). By SPECT/CT imaging, [^111^In]EDTA was mainly excluded from the brain cavity at 30 min after injection and cleared from blood by 4 h (Fig. S8a). The amount of [^111^In]EDTA measured in the brain was significantly lower than the values obtained for [^111^In]MWNT-Fab′ throughout the studied time points (Fig. S8b). These results confirmed that the radioactivity detected in the brain after [^111^In]MWNT-Fab′ injection originated from [^111^In]MWNT-Fab′ rather than free ^111^In which may have dissociated from MWNT-Fab′-DTPA after injection.

**Fig. 3:**
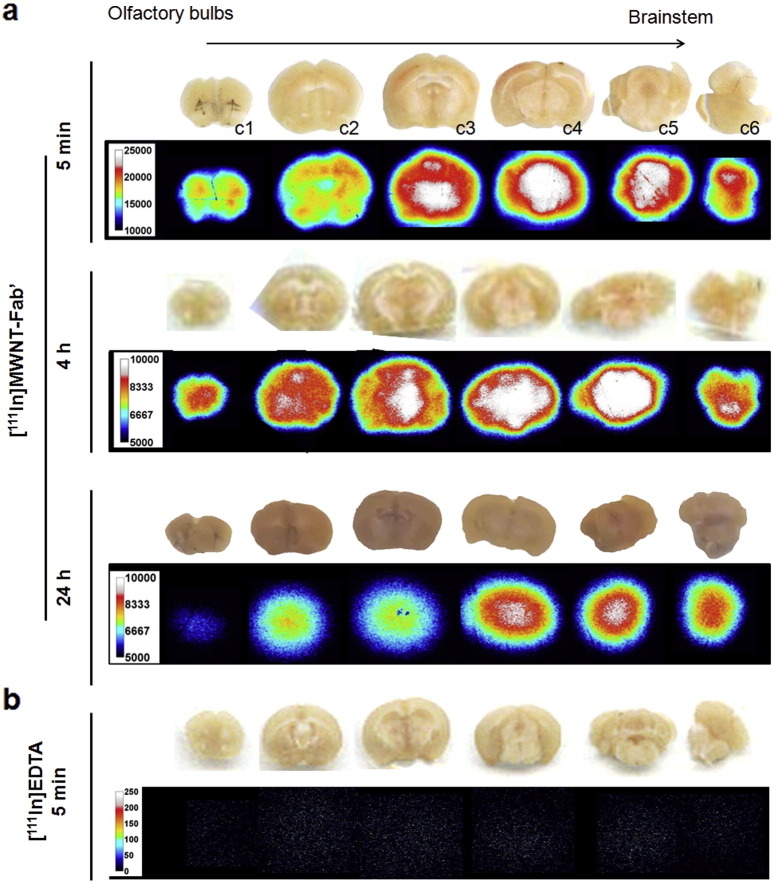
Autoradiographs of mouse brain after i.v. injection of [^111^In]MWNT-Fab′ or [^111^In]EDTA at different time points. Mice were i.v. injected with 5–7 MBq of (a) [^111^In]MWNT-Fab′ (50 μg) or (b) [^111^In]EDTA. Brains were harvested at 5 min, 4 h or 24 h after injection. In the case of [^111^In]EDTA, autoradiography was only performed at 5 min after injection. Brains were sectioned in coronal orientation from olfactory bulbs to brainstem in 2 mm thick sections (c1–c6).

Autoradiography of brain slices was also conducted to provide greater spatial resolution (μm range), allow sensitivity improvement by increasing the exposure time [29] and support semi-quantitative analysis of particle accumulation in different brain regions. Fig. 3a shows representative autoradiographs of [^111^In]MWNT-Fab′ from coronal brain slices (2 mm thick) at different time points after injection. The accumulation of [^111^In]MWNT-Fab′ in the midbrain area (sections c3–5) was clearly captured, with relatively lower radioactivity detected in brain cortex compared to the central thalamic region. Fig. 3b are autoradiographs of brain slices from the mice injected with [^111^In]EDTA containing an equivalent dose of radioactivity. The results confirmed that [^111^In]EDTA does not accumulate in the brain. It should be noted that the exposure times for different brains were slightly modified to achieve optimised image contrast output, allowing direct comparison between slices from the same brain at one time point, but not directly between brains at different time points.

To investigate further the heterogeneous brain distribution of [^111^In]MWNT-Fab′, *in situ* brain perfusion was used which replaced blood with physiological ringer perfusate containing [^111^In]MWNT-Fab′. The perfusate is constantly supplied *via* the left cardiac ventricle to the major arteries supplying the head, with outflow *via* the descending vena cava and right atrium. This allows control of the [^111^In]MWNT-Fab′ concentration reaching the brain which remains constant under steady flow conditions with no recirculation of the construct, and bypasses clearance to peripheral organs such as liver. In this case, reductions in brain uptake over time can be reliably attributed to clearance from the brain.

Accumulation of [^111^In]MWNT-Fab′ in different brain regions was analysed by γ-counting at the end of a perfusion period of 5, 10 or 30 min. As shown in Fig. S9a, the radioactivity in different brain regions increased when perfusion times increased from 5 min to 10 min. However, a reduction was detected when the perfusion lasted for 30 min. One possibility is that the brain intake of *f*-MWNTs became saturated after 10 min of steady administration so that no further entry to brain was permitted; however in that case we would expect brain uptake at 30 min to be similar to uptake at 10 min. Another possibility is that there is a clearance mechanism from the brain which equals or exceeds uptake after 10 min. The decreased radioactivity in different brain regions detected at 30 min could be a result of both factors, *i.e.*, saturation of brain entry and a clearance mechanism. Fig. S9b displays the % ID/g of tissue and no significant differences were observed between different brain regions at the same perfusion time. The results correlate well with the SPECT images at 0–30 min where the uptake of [^111^In]MWNT-Fab′ was more homogeneous throughout the entire brain (Fig. 2).

SPECT/CT imaging and autoradiography addressed the brain uptake of MWNT-Fab′-DTPA based on the detection of the radioisotope bound to the nanotubes. Direct imaging of MWNT-Fab′-DTPA using several state-of-art microscopic imaging techniques was further conducted and documented below with the attempt to visualise MWNT-Fab′-DTPA with greater spatial resolution in a label-free manner.

### 3.4 Histology and Raman imaging

The brain distribution of non-radiolabelled MWNT-Fab′-DTPA was assessed directly by light microscopy and Raman spectroscopy. For light microscopy, parafinised brain sections were stained with Nissl & Luxol Fast Blue (LFB) or Neutral Red. MWNT-Fab′-DTPA could be visualised as small black clusters in different brain regions with no abnormality in brain structure observed (arrow heads, Fig. S10).

**Fig. 4:**
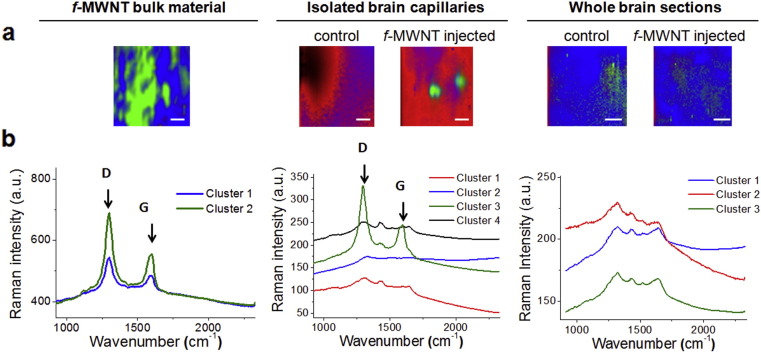
Raman microscopy of MWNT-Fab′-DTPA in mouse brain. Raman microscopy and clustering analysis were performed on MWNT-Fab′-DTPA, isolated brain capillaries or whole brain sections. Brain samples were from control mice or mice i.v. injected with MWNT-Fab′-DTPA (200 μg, 5 min). Raman microscopic images are shown in (a), generated using cluster analysis. Clusters exhibiting similar Raman spectra are represented with the same colour. The corresponding average Raman spectra for each of the clusters are shown in (b). Scale bars are 13 μm. D and G bands characteristic of MWNT could be only detected in treated capillaries but not in control capillaries, untreated or treated whole brain sections.

CNTs can be imaged *in vivo* due to their inherent optical properties by Raman spectroscopy, NIR fluorescence microscopy and photoacoustic imaging [41], [42], [43], [44]. In this study, Raman spectroscopy was employed to image MWNT-Fab′-DTPA in the brain (Fig. 4). MWNT-Fab′-DTPA bulk material (left panel) and samples of isolated brain capillaries (middle panel) or whole brain sections from mice injected with MWNT-Fab′-DTPA (right panel) were prepared along with samples from un-injected mice as controls. Fig. 4a presents Raman microscopic images generated using cluster analysis. Clusters exhibiting similar Raman spectra are presented in the same colour. The corresponding average Raman spectra from different clusters are shown in Fig. 4b with the same colour codes. The typical D (1309 cm^− 1^) and G (1599 cm^− 1^) bands, characteristic of MWNTs, were clearly detected in the Raman spectra of MWNT-Fab′-DTPA bulk material. In the isolated capillaries, 4 types of clusters with different Raman spectra were identified. Clusters 1, 2 and 4 were related to tissue background as they were also detected in control capillaries in the absence of MWNT-Fab′-DTPA. Cluster 3, exhibiting the typical D and G bands of MWNTs, was detected only in brain capillaries from mice following i.v. MWNT-Fab′-DTPA treatment. Due to sensitivity limitations of the technique, MWNT-Fab′-DTPA could not be detected in whole brain sections, whose spectra obtained were identical to those of control brains. This is expected for two reasons; first, MWNT-Fab′-DTPA achieved ~ 4 fold higher uptake in capillaries compared with brain parenchyma; secondly, the Raman microscope employed is of modest sensitivity and whole brain sections were only 10 μm thick with less total tissue compared to the capillary sample which was concentrated prior to imaging.

### 3.5 Multi-photon luminescence imaging and lifetime microscopy of MWNT-Fab′-DTPA in whole brain slices

**Fig. 5:**
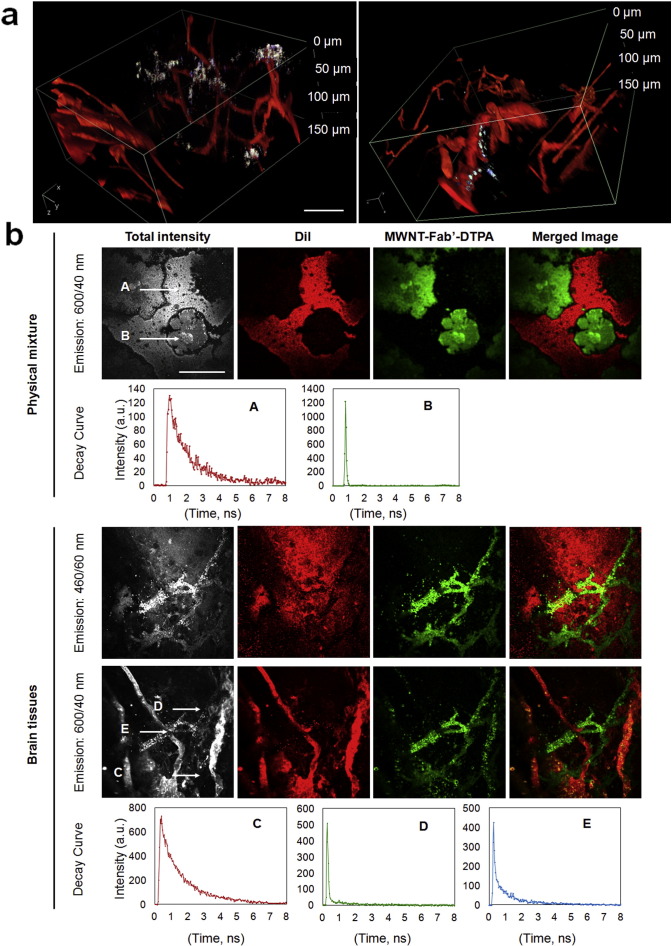
Multi-photon luminescence imaging and lifetime microscopy of brain slices from mice injected with MWNT-Fab′-DTPA. (a) 3D reconstruction of multi-photon luminescence imaging of MWNT-Fab′-DTPA in brain slices (z-step: 1 μm, number of optical sections: 150; λ_excitation_ = 950 nm). Blood vessels appear in red (Dil stained) while MWNT-Fab′-DTPA appears in white; (b) lifetime microscopy of MWNT-Fab′-DTPA & DiI physical mixture, and MWNT-Fab′-DTPA in brain slices by multi-photon excitation. From left: total multi-photon fluorescence intensity (grey), intensity contribution from long lifetimes (DiI, red), contribution from short lifetimes (MWNT-Fab′-DTPA, green), and merged image. Lifetime measurements of positions A–E are displayed below the multiphoton images. A, C and B, D correspond to DiI and MWNT-Fab′-DTPA, respectively, while E corresponds to the position where both were present. Mice were i.v. injected with MWNT-Fab′-DTPA (200 μg) and brains were isolated at 1 h after injection. Mice were perfused with DiI and 4% PFA at sacrifice. Brains were sectioned into 1 mm thick slices. Scale bar: 50 μm.

Multi-photon luminescence microscopy has been previously used to image carbon materials *in vivo* [45] and two-photon photoluminescence of SWNT has also been described [46]. In our recent study, the multi-photon excitation property of *f*-MWNTs was investigated and imaging parameters were optimised to visualise *f*-MWNTs in cells and parafinised tumour section after local administration [47]. The present study utilised multi-photon excitation microscopy to image MWNT-Fab′-DTPA in whole brain for the first time. Mice were i.v. injected with MWNT-Fab′-DTPA and perfused with DiI to stain blood vessels one hour later [28] at sacrifice. 3D reconstructed images of brain slices are shown in Fig. 5a and Movies S1 and S2. DiI-stained blood vessels are shown in red. MWNT-Fab′-DTPA exhibited broad photoluminescence spectra (λ_excitation_ = 950 nm) so it could be imaged in blue, green and red channels, appearing white in the merged images. The corresponding maximum intensity projection images with signals detected from separate channels are shown in Fig. S11. Lifetime microscopy was then performed to distinguish the photoluminescence from MWNT-Fab′-DTPA and the fluorescence from DiI (Fig. 5b). Separate components of the overall multiphoton-excited signals from physically-mixed DiI and MWNT-Fab′-DTPA dispersion were differentiated using a convoluted Gaussian and mono-exponential fitting model:

$$\text{Intensity}\left(t\right)=\text{Offset}+\int \mathrm{IRF}\left(t^{\prime }\right)*\left[\mathrm{PL}\left(t-t^{\prime }\right)+\text{Fluorescence}\;\left(t-t^{\prime }\right)\right]dt^{\prime }\;$$

The phenomenon of the fast relaxation measured from MWNT-Fab′-DTPA is expected as CNTs are known to possess very short PL lifetimes [48], whereas DiI, being an organic fluorophore, displayed a longer fluorescence lifetime > 1 ns [49]. The distinct differences in lifetimes of DiI and MWNT-Fab′-DTPA permit the distinction between DiI and MWNT-Fab′-DTPA signals. Individual lifetime images of the two components were clearly resolved by lifetime microscopy, providing evidence of the presence of MWNT-Fab′-DTPA in brain tissues.

### 3.6 Transmission electron microscopy (TEM)

**Fig. 6:**
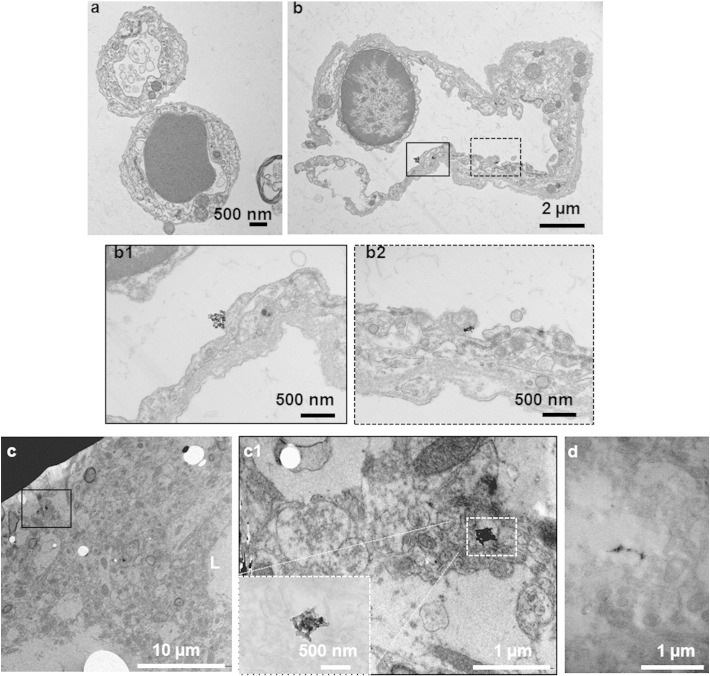
TEM studies of MWNT-Fab′-DTPA in whole brain sections and isolated brain capillaries. Electron micrographs of brain capillary extracts from (a) control mice; (b) MWNTs-Fab′-DTPA injected mice. MWNT-Fab′-DTPA were observed in few images, illustrated in the solid and dashed rectangular insets to below b1 and b2. Electron micrographs of electron dense MWNT-Fab′-DTPA were more readily observed in whole brain sections (c). An adjacent capillary was observed (L indicates the lumen of a capillary). The black rectangular inset identifying MWNT-Fab′-DTPA clusters is shown in (c1) under a higher magnification and also with enhanced contrast (white dashed rectangular inset). (d) Is another example of electron micrograph of MWNT-Fab′-DTPA observed in brain sections. Mice were i.v. injected with MWNT-Fab′-DTPA (200 μg) and were perfused with 0.1 M cacodylate buffer containing 2.5% glutaraldehyde at 5 min after injection. The isolated brains were immersed in the perfusate for 24 h at 4 °C before further process for TEM imaging with detailed methods described in the Materials and methods section.

TEM is one of the best techniques at present to depict the morphology of single, isolated MWNT and the surrounding biological environment at the nanoscale level [50]. It was used to verify MWNT-Fab′-DTPA brain parenchyma uptake. TEM was first conducted on isolated brain capillaries, showing the association of MWNT-Fab′-DTPA with the brain microvasculature. Two brain capillaries from control mice are shown in Fig. 6a, each with a single endothelial cell forming the vessel wall and with one capillary containing a red blood cell. Brain capillaries isolated from mice injected with MWNT-Fab′-DTPA contained few clusters of MWNT-Fab′ (Fig. 6b), observed residing on the luminal side of an endothelial cell (solid and dashed rectangular insets, Fig. 6b1 & b2). MWNT-Fab′-DTPA was also observed in whole brain sections (Fig. 6c, d, and the higher magnification image in 6c1), probably in brain parenchyma as being distant from a brain capillary (the lumen indicated as L at the edge of Fig. 6c).

## 4 Conclusions

The kinetic distribution of ^111^In-labelled MWNT-Fab′-DTPA within the brain environment was investigated in this study by quantitative γ-scintigraphy, SPECT/CT imaging and autoradiography after i.v. injection in mice. The localization of MWNT-Fab′-DTPA with brain capillaries, demonstrated *in vivo* using γ-counting (Fig. 1c) and TEM (Fig. 6), suggests that the entry of MWNTs into the brain occurs *via* its endothelium. Significant brain parenchyma to blood ratios during 1–4 h after injection (Fig. 1d) indicate the ability of MWNT-Fab′-DTPA to circumvent the BBB. Taking advantage of the intrinsic optical properties of MWNT, MWNT-DTPA-Fab′ was imaged in brain in a label-free manner by Raman microscopy and multi-photon luminescence microscopy with satisfactory resolution and sensitivity (Fig. 4, Fig. 5). Lifetime microscopy based on multi-photon excitation further confirmed the existence of MWNT-Fab′-DTPA in the brain. This study opens new opportunities to use *f*-MWNT as a delivery system for therapeutic or diagnostic application to CNS. Studies using *f*-MWNT conjugated with a brain targeting ligand is currently undertaking.

The following are the supplementary data related to this article.

Supplementary material.

Movies S1 & S2 3D reconstruction of multi-photon luminescence imaging of MWNT-Fab′-DTPA in brain slices. Mice were i.v. injected with MWNT-Fab′-DTPA (200 μg) and brains were isolated at 1 h after injection. Mice were perfused with DiI and 4% PFA at sacrifice. Brains were sectioned into 1 mm thick slices (z-step: 1 μm, number of optical sections: 150; λ_excitation_ = 950 nm). Blood vessels appear in red (Dil stained) while *f*-MWNT appears in white.
