# Supplementary material for: Kinetics of functionalised carbon nanotube distribution in mouse brain after systemic injection: Spatial to ultra-structural analyses
Source: J Control Release. 2016 Feb 28;224:22–32. doi: 10.1016/j.jconrel.2015.12.039 (PMC4756275; doi:10.1016/j.jconrel.2015.12.039)
Supplement: Supplementary file 1 — Supplementary material. [file mmc1.pdf]

Kinetics of functionalised carbon nanotube distribution in mouse brain after intravenous injection: Spatial to ultra-structural analyses

Julie T.-W. Wang[1], Noelia Rubio[1], Houmam Kafa[1], Enrica Venturelli[2], Chiara Fabbro[3], Cécilia Ménard-Moyon[2], Tatiana Da Ros[3], Jane K. Sosabowski[4], Alastair D. Lawson[5], Martyn K. Robinson[5], Maurizio Prato[3], Alberto Bianco[2], Frederic Festy[6], Jane E. Preston[1], Kostas Kostarelos[7]*, and Khuloud T. Al-Jamal[1]*

[1] Institute of Pharmaceutical Science, Faculty of Life Sciences & Medicine, King's College London, London SE1 9NH, UK

[2] CNRS, Institut de Biologie Moléculaire et Cellulaire, Laboratoire d'Immunopathologie et Chimie Thérapeutique, Strasbourg F-67000, France

[3] Dipartimento di Scienze Chimiche e Farmaceutiche, Università di Trieste, Trieste 34127, Italy

[4] Centre for Molecular Oncology, Barts Cancer Institute, Queen Mary University of London, London EC1M 6BQ, UK

[5] UCB Celltech, Slough, Berkshire SL1 3WE, UK

[6] Tissue Engineering and Biophotonics, Dental Institute, King's College London, London SE1 9RT, UK

[7] Nanomedicine Laboratory, UCL School of Pharmacy, University College London, Brunswick Square, London, UK

* Corresponding authors. E-mails: khuloud.al-jamal@kcl.ac.uk; k.kostarelos@ucl.ac.uk

Supplementary materials and methods

Examination of human MUC1 expression in mouse brain by Western blot analysis

Isolated mouse brains were kept on ice and homogenised immediately after adding ice-cold RIPA lysis buffer (150 mM NaCl, 0.1 %Triton X-100, 0.5 % sodium deoxycholate, 0.1 % SDS, 50 mM Tris-HCl, pH 8) containing protease inhibitor using an electric homogeniser. After 30 min - 1 h incubation, homogenates were centrifuged at 17949 x *g* for 30 min and the supernatants were collected. Human breast cancer cells, MCF-7 (ATCC®, HTB-22™), are known to express high level of MUC1 and were used as a positive control. Cells were cultured in DMEM medium (Life Technologies, UK) supplemented with 10% FBS, 50 U/ml penicillin, 50 µg/ml streptomycin and 1 % L-glutamine in a T75 flask at 37 °C in a 5 % CO_2 incubator. When reaching 70-80% confluency, cells were rinsed twice with ice-cold PBS, incubated with 500 µl RIPA lysis buffer containing protease inhibitor and scrapped out. Cell proteins were collected after centrifugation as described. The protein concentrations from brain and cell samples were determined by BCA assay. Proteins (30 µg) from each sample was resolved in 8 % SDS-PAGE gels and transferred to Hybond ECL nitrocellulose membranes (GE Healthcare, UK). The membrane was blocked in 3% bovine serum albumin (Sigma-Aldrich, UK) at room temperature for 1 h, incubated with humanised anti-human MUC1 antibody (hCTM01 IgG, UCB Celltech, UK) at 10 µg/ml overnight on ice and incubated with the secondary antibody, horseradish peroxidase linked anti-human antibody (Cell Signalling Technology, USA) at 1:1000 dilution for 2 h at room temperature. Protein bands were detected using chemiluminescent kits (Immun-StarTM Chemiluminescent Kit, BioRad, UK) and imaged and quantitatively analysed using ChemiDoc MP imaging system and Image Lab software (BioRad, UK).

Supplementary Figures

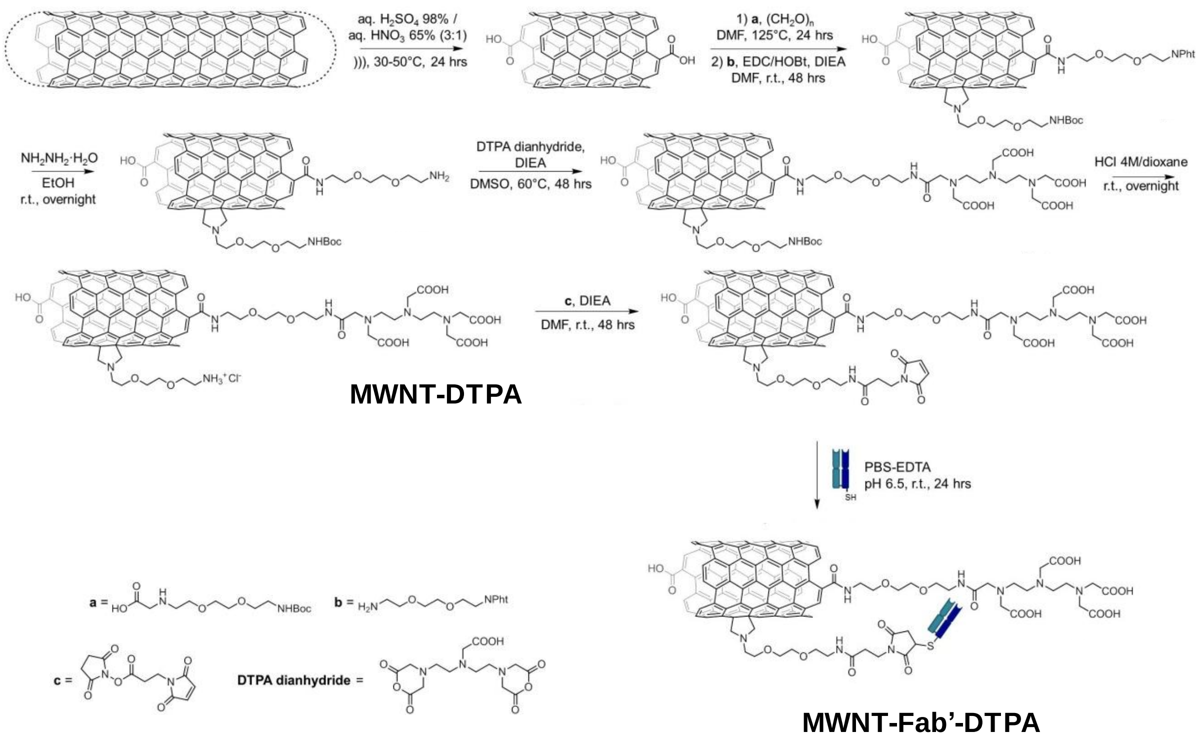

Fig. S1 Scheme of the synthesis of MWNT-Fab'-DTPA (modified from reference (*23*)).

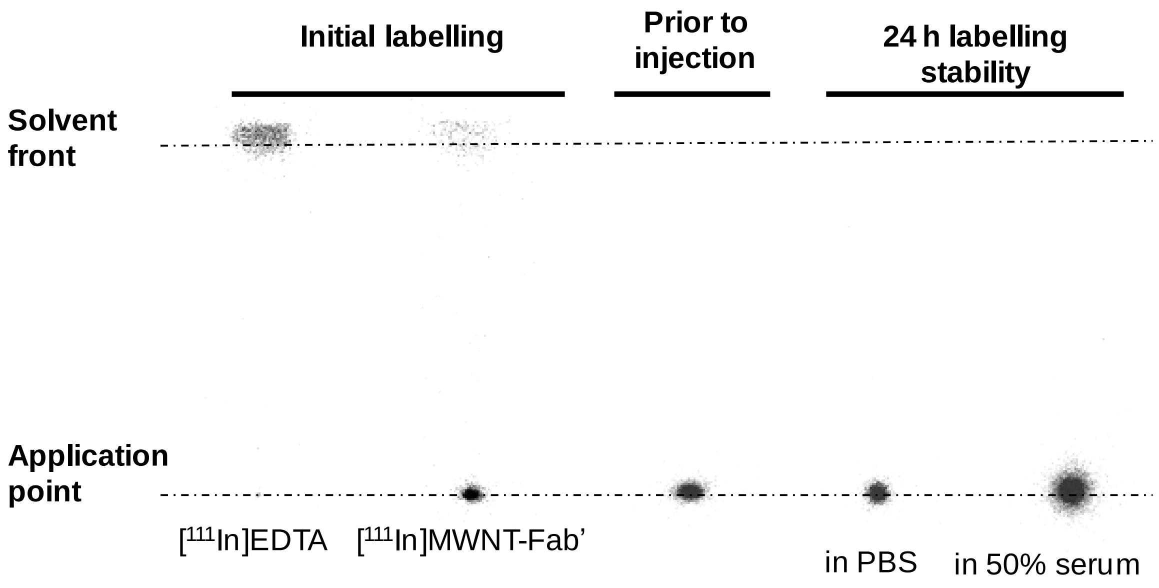

Fig. S2 Radiolabelling efficiency of MWNT-Fab'-DTPA and labelling stability. TLC analyses of [^{111}In]MWNT-Fab' immediately after radio-labeling reaction and quenching by EDTA, prior to injection, and radiolabelling stability after an incubation in PBS or 50 % serum (serum:PBS, 1:1) at 37 °C for 24 h.

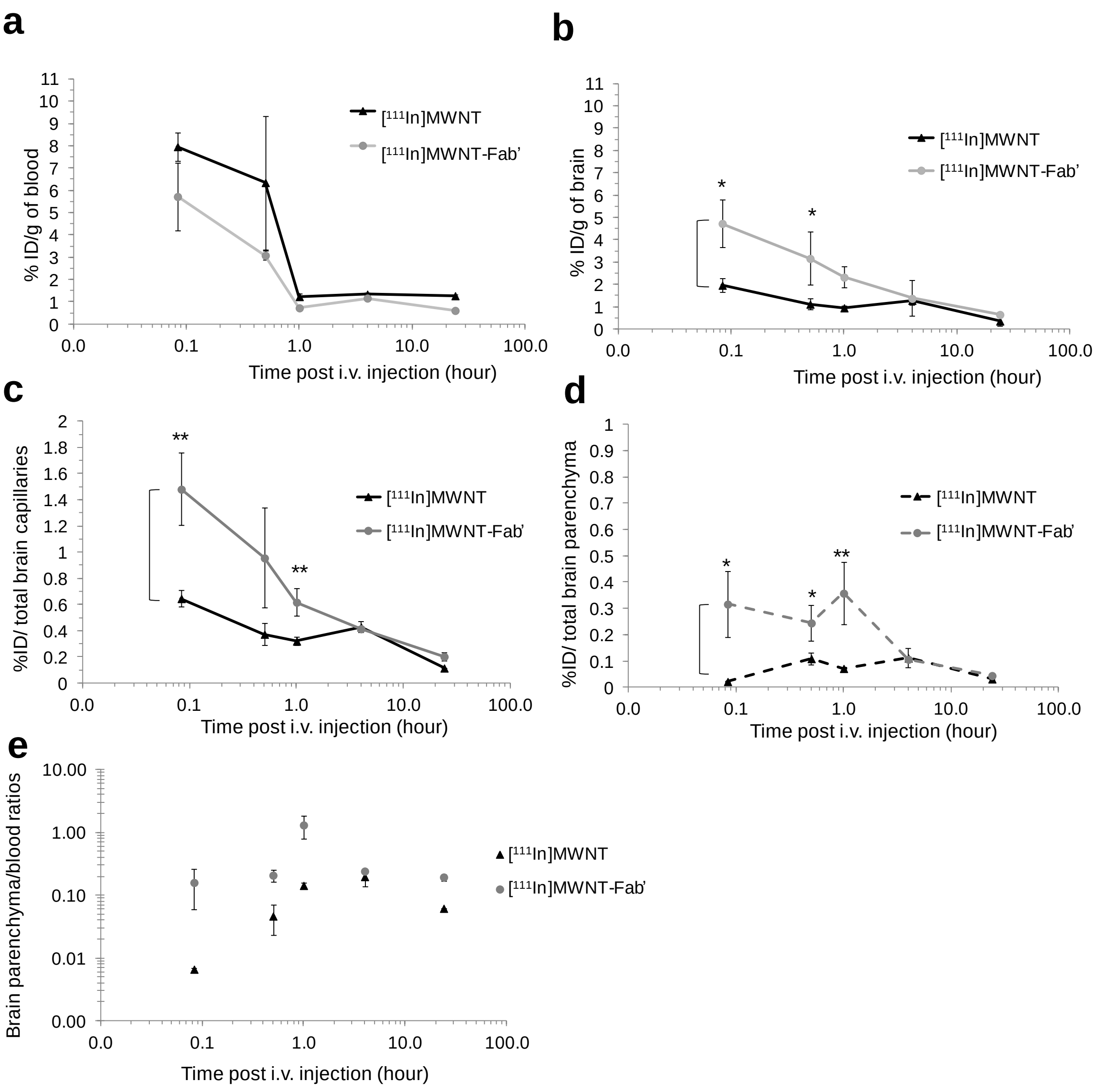

Fig. S3: Blood circulation and brain uptake of [^{111}In]MWNT and [^{111}In]MWNT-Fab' after i.v. injection at different time points by γ-scintigraphy. (a, b) % ID/g of blood and brain; **(c, d)** disposition of [^{111}In]MWNT and [^{111}In]MWNT-Fab' (%ID) in the brain capillaries and parenchyma respectively after the capillary depletion treatment; **(e)** brain parenchyma to blood ratio analysis. Mice were i.v. injected with 50 µg of [^{111}In]MWNT or [^{111}In]MWNT-Fab'. Brain parenchyma/blood ratios were calculated based on the concentrations in the brain parenchyma and in the blood (%ID/g). Results are expressed as mean ± S.D. (n=4). Statistical analysis was performed using One-Way ANOVA (*$p \leq 0.05$; **$p \leq 0.01$).

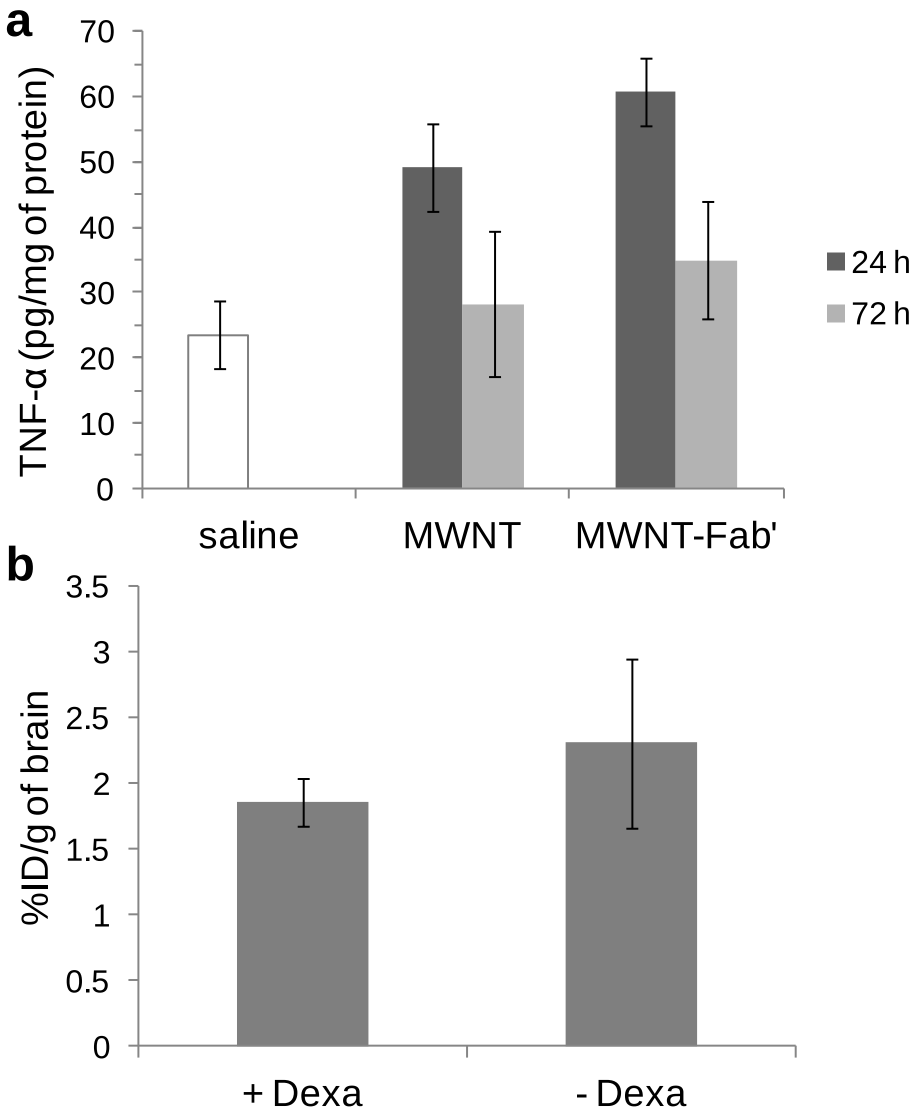

Fig. S4 Assessment of inflammatory response after i.v. administration of [^{111}In]MWNT or [^{111}In]MWNT-Fab'. (a) assessment of TNF-α levels in mouse brain at 24 or 72 h after injection of 50 μg of [^{111}In]MWNT-Fab' **(b)** brain uptake of [^{111}In]MWNT-Fab' with or without pre-treatment with dexamethasone (Dexa). TNF-α levels in mouse brain were examined by ELISA. Mice were injected with dexamethasone (Dexa, 10 mg/kg i.p.). One hour later, control mice and Dexa-treated mice were i.v. injected with [^{111}In]MWNT-Fab' and brains isolated after a further 1 h for γ-scintigraphy. Cardiac perfusion to wash out residual blood containing *f*-MWNT was performed before tissue harvesting. Results are expressed as the mean TNF- α levels in brain (pg/mg of protein) and %ID/g of brain ± S.D., respectively (n=3).

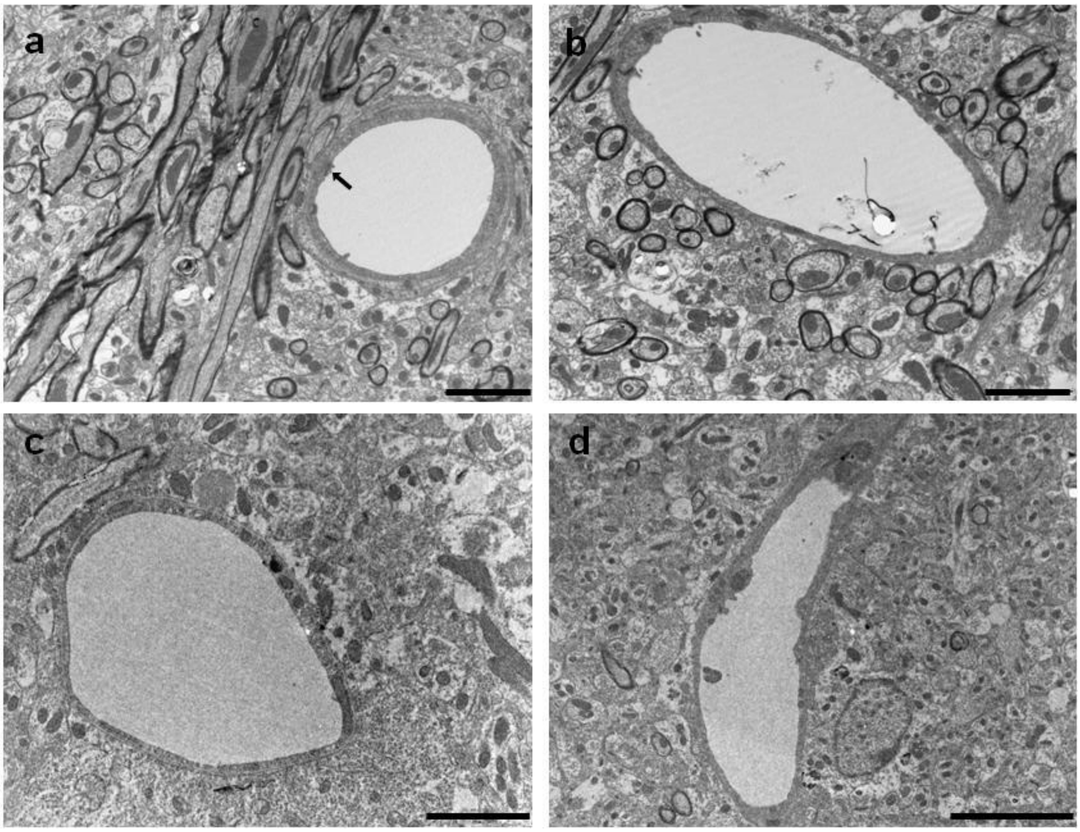

Fig. S5: The state of brain capillaries following exposure to MWNT-DTPA-Fab'. Electron micrographs stained with uranyl acetate and lead citrate **(a-b)** or uranyl acetate only **(c-d)** showing intact brain capillaries following exposure to MWNT-DTPA-Fab'. Tight junction assembly (black arrow) appear intact with no apparent pathological damage to the endothelial cells. Mice were i.v. injected with 200 µg MWNT-Fab'-DTPA and brains were harvested at 5 min after cardiac perfusion and fixation with glutaldehyde solution. Scale bars are 2 µm in **(c)** and 5 µm for the rest of the graphs.

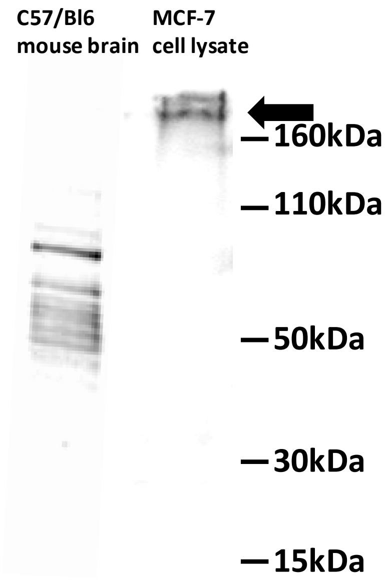

Fig. S6 Western blot analysis of MUC1 protein expression from extracts of mouse brain and human breast cancer MCF-7 cells. The lack of MUC1 protein in mouse tissue or the lack of cross-reactivity of mouse brain tissue with anti-human MUC1 antibody was confirmed by Western blot analysis. Bands of MUC 1 protein (~170 kDa) were observed in human breast cancer cell line MCF-7 (black arrow) which is known to over-express MUC1 proteins. No MUC 1 protein was detected from C57/Bl6 mouse brain homogenates.

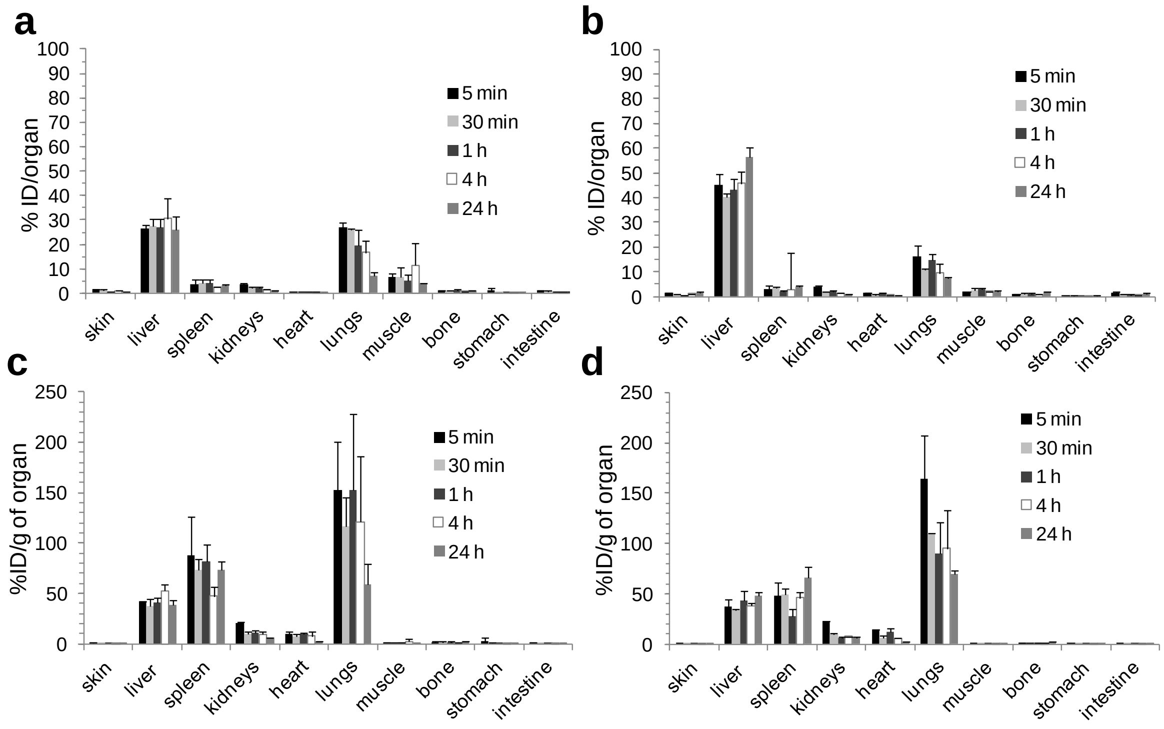

Fig. S7 Biodistribution of [^{111}In]MWNT and [^{111}In]MWNT-Fab' in organs other than brain and blood at different time points after i.v. injection. (a, b) % ID/organ **(c, d)** % ID/g of organ of [^{111}In]MWNT and [^{111}In]MWNT-Fab', respectively. Mice were i.v. injected with 50 µg of [^{111}In]MWNT or [^{111}In]MWNT-Fab'. Tissues were excised after whole body cardiac perfusion. Results are expressed as mean ± S.D. (n=4).

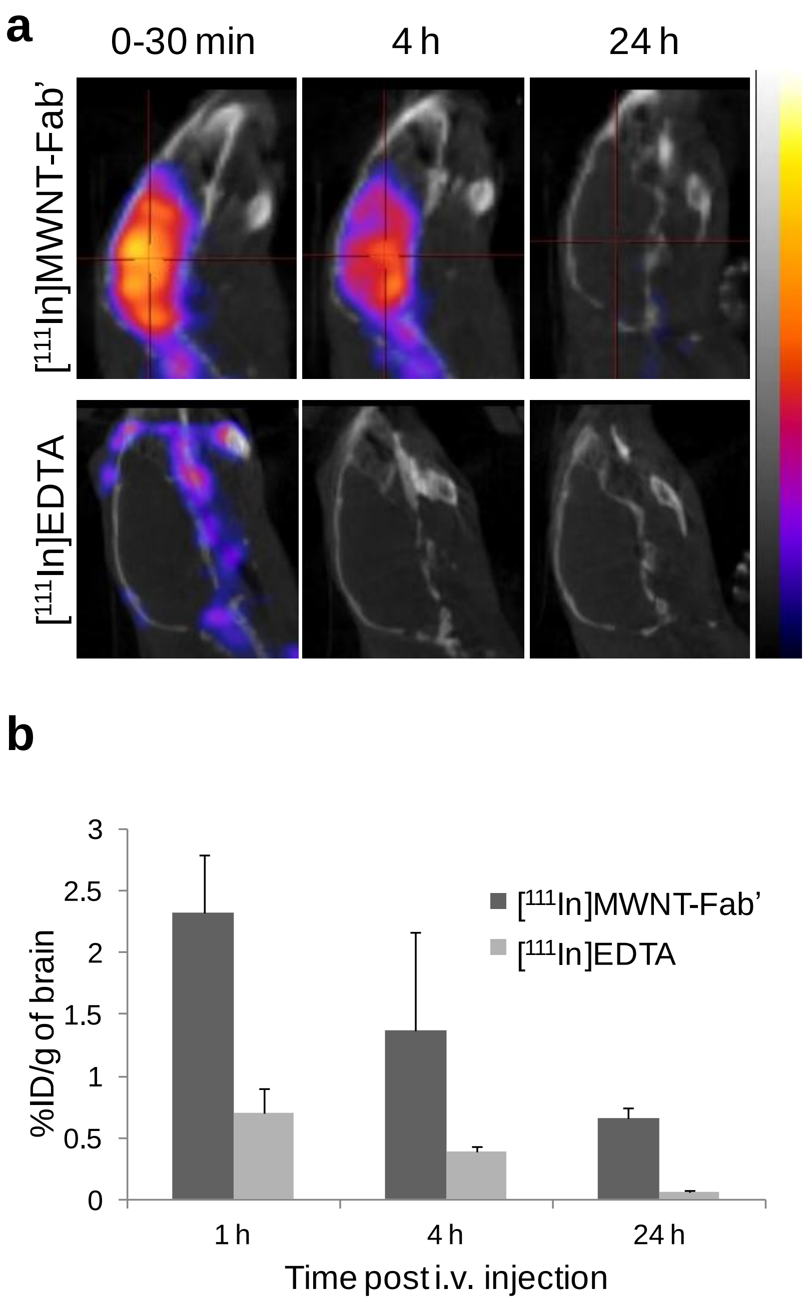

Fig. S8 Comparison of the brain uptake of [^{111}In]MWNT-Fab' and [^{111}In]EDTA radiolabel alone at 0-30min, 4 h and 24 h after i.v. injection. (a) Sagittal views of SPECT/CT imaging of mice brain; **(b)** % ID/g of brain. Mice were i.v. injected with 50 µg of [^{111}In]MWNT-Fab'. Cardiac perfusion to wash out residual blood MWNT was performed for mice injected with [^{111}In]MWNT-Fab' but not for [^{111}In]EDTA . Results are expressed as mean ± S.D. (n=4).

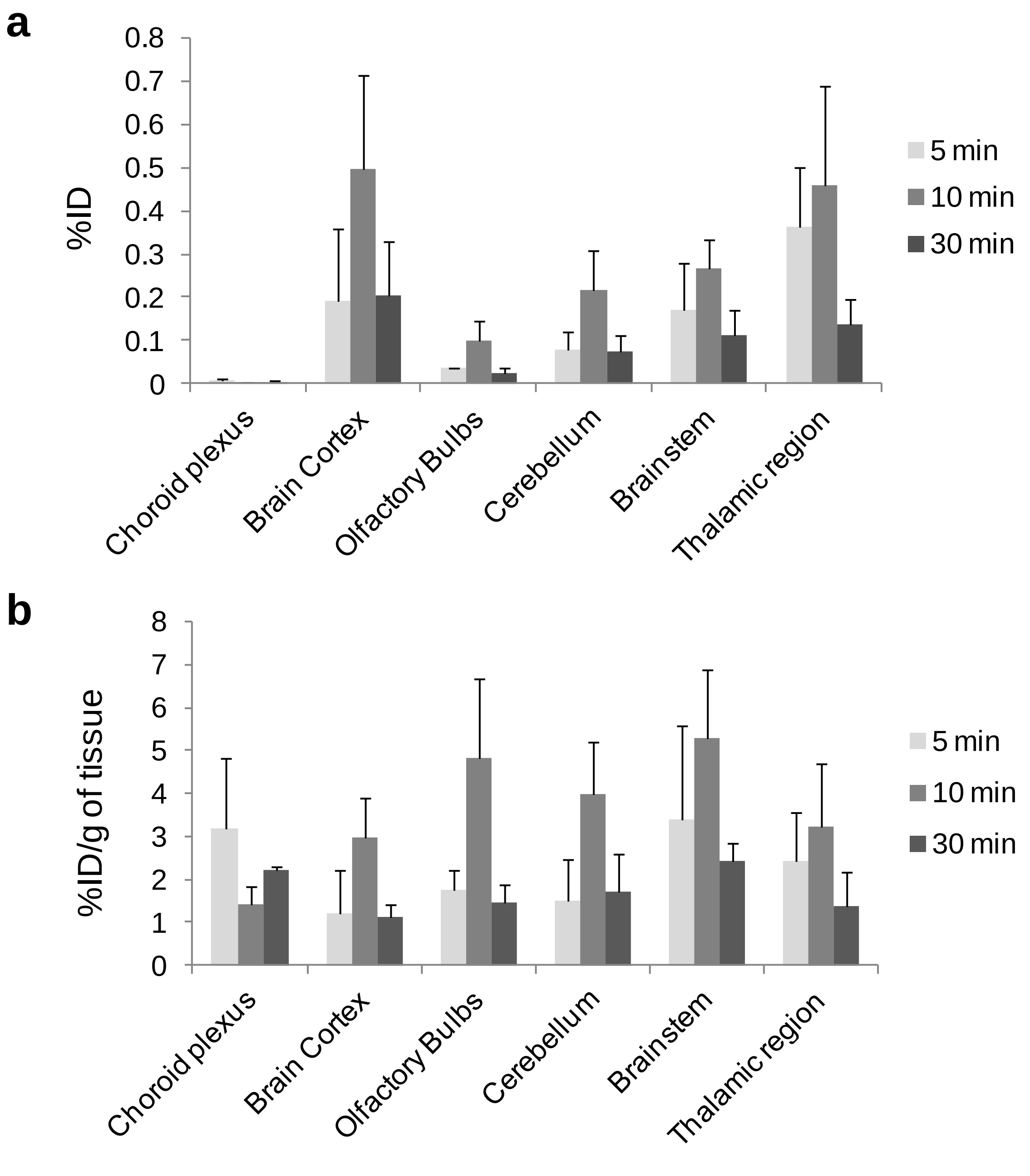

Fig. S9 Uptake of [^{111}In]MWNT-Fab' in different brain regions after *in situ* brain perfusion for different periods of time. (a) % ID/brain region **(b)** % ID/g of tissue. Mice were perfused continuously with [^{111}In]MWNT-Fab' (0.4 µg/ml) at a constant rate of 5 ml/min. Results are expressed as mean ± S.D. (n=3).

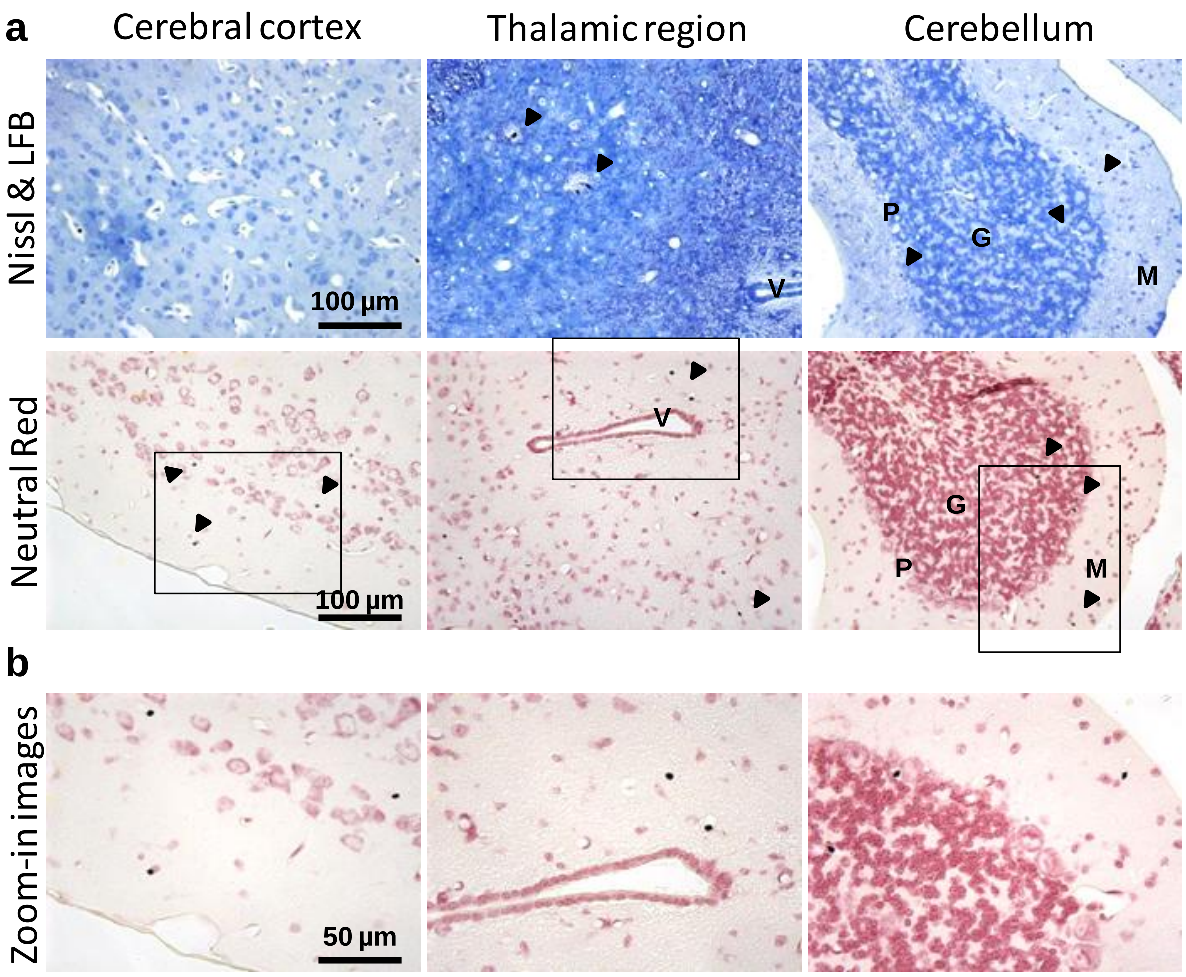

Fig. S10 Histological examination of mouse brain after i.v. injection of MWNT-Fab'-DTPA. (a) Light microscopy images from different brain regions stained with Nissl & LFB or Neutral Red. **(b)** Neutral Red stained brain images with higher magnification. Mice were i.v. injected with 200 µg MWNT-Fab' and brains were harvested at 5 min after cardiac perfusion to remove vascular construct. Arrow heads indicate the presence of small clusters of MWNT-Fab'-DTPA. V: blood vessels; G: granular layer; M: Molecular layer; P: Purkinje cells.

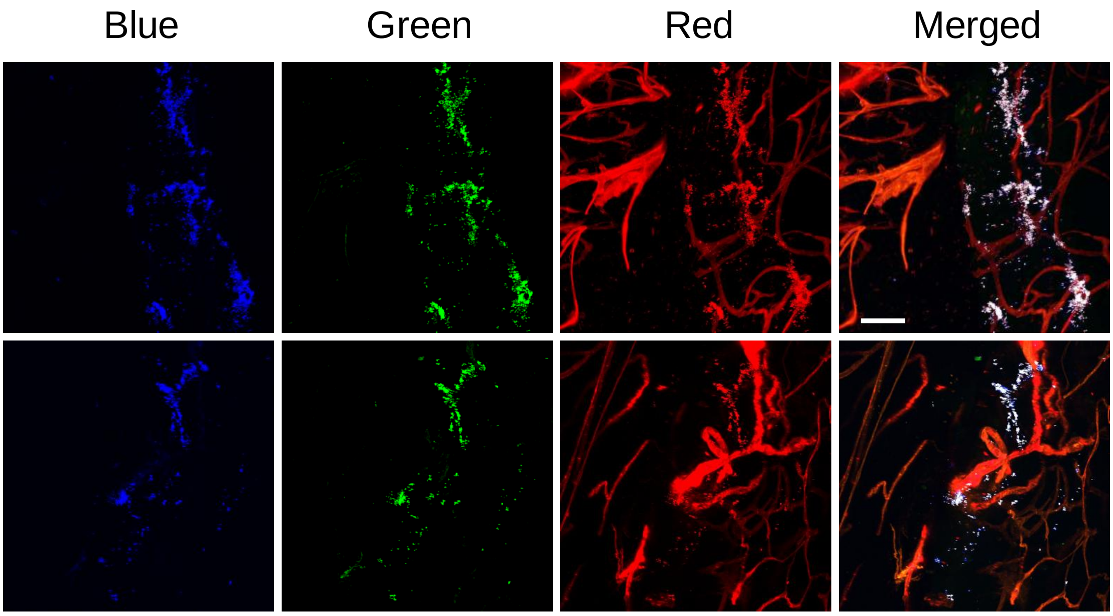

Fig. S11 Maximum intensity projection images of MWNT-Fab'-DTPA in DiI-perfused whole brain produced with multi-photon luminescence microscopy. Mice were i.v. injected with MWNT-Fab'-DTPA (200 µg) and brains were isolated at 1h after injection. Mice were perfused with DiI to stain blood vessels (red) and 4 % PFA at sacrifice. Brains were sectioned into 1 mm thick slices. Images were acquired with multi-photon excitation at 950 nm capturing MWNT-Fab'-DTPA images in the blue, green and red channels, appearing white in the merged images. Scale bar: 50 µm.

Movies S1 & S2: 3D reconstruction of multi-photon luminescence imaging of MWNT-Fab'-DTPA in brain slices. Mice were i.v. injected with MWNT-Fab'-DTPA (200 μg) and brains were isolated at 1h after injection. Mice were perfused with DiI and 4 % PFA at sacrifice. Brains were sectioned into 1 mm thick slices (z-step: 1 μm, number of optical sections: 150; $\lambda_{excitation}$ = 950 nm). Blood vessels appear in red (DiI stained) while *f*-MWNT appears in white.

References and Notes

23. J. T. W. Wang, C. Fabbro, E. Venturelli, C. Ménard-Moyon, O. Chaloin, T. Da Ros, L. Methven, A. Nunes, J. K. Sosabowski, S. J. Mather, M. K. Robinson, J. Amadou, M. Prato, A. Bianco, K. Kostarelos, K. T. Al-Jamal, The relationship between the diameter of chemically-functionalized multi-walled carbon nanotubes and their organ biodistribution profiles *in vivo*. *Biomaterials* **35**, 9517 (2014).
