# Supplementary figures and images for: Kinetics of functionalised carbon nanotube distribution in mouse brain after systemic injection: Spatial to ultra-structural analyses
Source: J Control Release. 2016 Feb 28;224:22–32. doi: 10.1016/j.jconrel.2015.12.039 (PMC4756275; doi:10.1016/j.jconrel.2015.12.039)

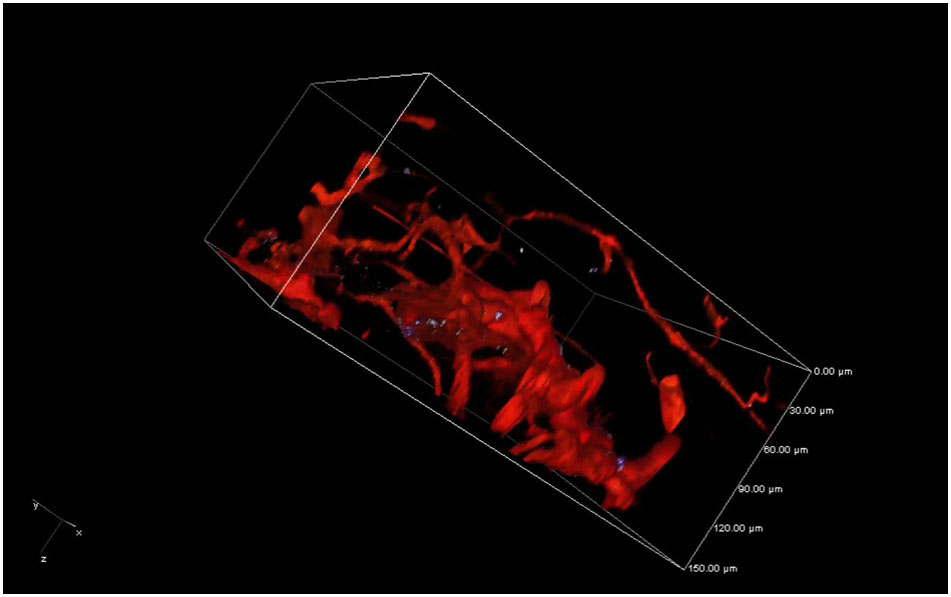

Supplement: Movies S1 & S2 — 3D reconstruction of multi-photon luminescence imaging of MWNT-Fab′-DTPA in brain slices. Mice were i.v. injected with MWNT-Fab′-DTPA (200 μg) and brains were isolated at 1 h after injection. Mice were perfused with DiI and 4% PFA at sacrifice. Brains were sectioned into 1 mm thick slices (z-step: 1 μm, number of optical sections: 150; λexcitation = 950 nm). Blood vessels appear in red (Dil stained) while f-MWNT appears in white. [file mmc2.jpg]

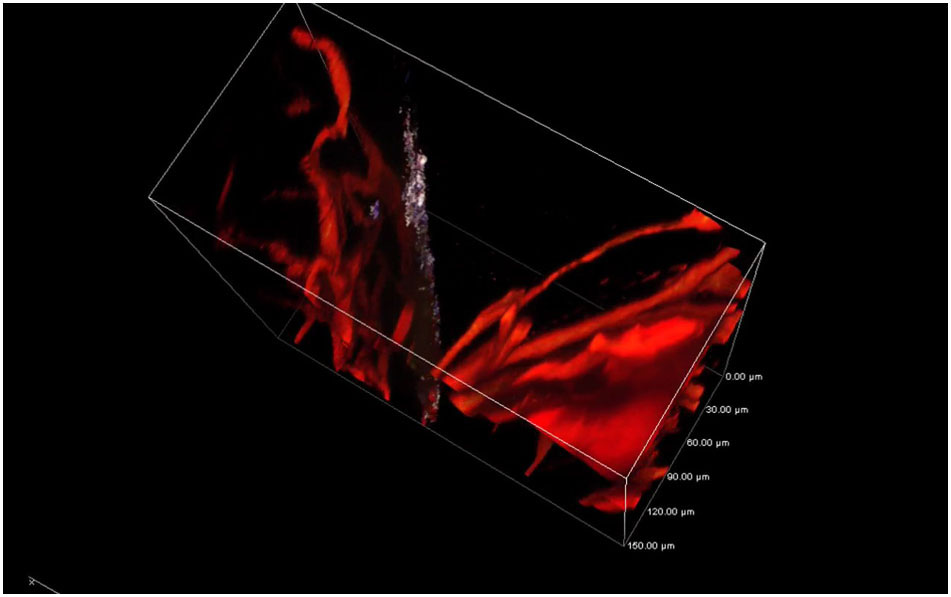

Supplement: Movies S1 & S2 — 3D reconstruction of multi-photon luminescence imaging of MWNT-Fab′-DTPA in brain slices. Mice were i.v. injected with MWNT-Fab′-DTPA (200 μg) and brains were isolated at 1 h after injection. Mice were perfused with DiI and 4% PFA at sacrifice. Brains were sectioned into 1 mm thick slices (z-step: 1 μm, number of optical sections: 150; λexcitation = 950 nm). Blood vessels appear in red (Dil stained) while f-MWNT appears in white. [file mmc3.jpg]
